# Demographic and Parental Factors Associated With Developmental Outcomes in Children With Intellectual Disabilities

**DOI:** 10.3389/fpsyg.2019.00872

**Published:** 2019-04-24

**Authors:** Rosa Vilaseca, Magda Rivero, Rosa M. Bersabé, María-José Cantero, Esperanza Navarro-Pardo, Clara Valls-Vidal, Fina Ferrer

**Affiliations:** ^1^Department of Cognition, Development and Educational Psychology, University of Barcelona, Barcelona, Spain; ^2^Department of Psychobiology and Methodology of the Behavioral Sciences, University of Málaga, Málaga, Spain; ^3^Department of Developmental and Educational Psychology, University of Valencia, Valencia, Spain; ^4^Psychology, Abat Oliba CEU University, Barcelona, Spain

**Keywords:** children with intellectual disabilities, child development, parental anxiety, parental depression, parental stress, family functioning, positive parenting

## Abstract

The aim of the study was to examine the relation between demographic variables, parental characteristics, and cognitive, language and motor skills development in children with intellectual disabilities (ID). A sample of 89 children with ID, aged 20–47 months, completed the Bayley Scales of Infant Development to measure cognitive, motor, and linguistic development. Parents were administered questionnaires about demographic information and parental anxiety, depression, parental stress, conjugality and familial functioning. Parenting behaviors (affection, responsiveness, encouragement, and teaching) were observed using the Spanish version of PICCOLO (Parenting Interactions with Children: Checklist of Observations Linked to Outcomes). A bivariate analysis showed that cognitive development in infants was significantly related to the mother’s and father’s responsiveness, and to the father’s teaching scores. Infant language development was related to a variety of maternal factors (educational level, anxiety, depression, maternal responsiveness) and to the father’s teaching scores. None of the factors were statistically related to child motor development. A multivariate regression analysis indicated that children’s cognitive development can be predicted by a linear combination of maternal responsiveness and paternal teaching scores. Language development can be predicted by a linear combination of maternal anxiety and responsiveness, and paternal teaching scores. The present study provides evidence of the importance of paternal involvement for cognitive and language development in children with intellectual disabilities, and contributes to the increasing literature about fathering. Gaining knowledge about parental contributions to children’s development is relevant for improving positive parenting in early intervention programs.

## Introduction

The influence of family context on children’s development has received increasing attention in recent years (Cabrera et al., 2011; Velasco et al., 2014; Chiang et al., 2015; Barreto et al., 2017), but understanding and explaining how this effect is exerted is a complex task.

In children with intellectual disability (ID), the slower rate of development affects their learning and their interaction and communication with others. Risk factors identified in children with ID include lower IQ, poorer communication skills, more medical illness, presence in some cases, of autistic symptomatology and difficulties in social interaction or behavior problems (Hauser-Cram et al., 2001).

### Sociodemographic Factors and Children’s Developmental Outcomes

Among the familial and demographic variables related to developmental outcomes, socioeconomic status (SES) has received considerable attention, usually in families with children of normative development (Sohr-Preston et al., 2013; Roubinov and Boyce, 2017). Among other variables, SES includes family income and parents’ educational level. In this respect, higher levels of parental education have been consistently associated with better developmental outcomes in children (Steinberg, 2001; Tamis-LeMonda et al., 2009). In contrast, a lower family SES is associated with a higher risk of delayed childhood development (Cheng, 2003; Emerson et al., 2006; Emerson and Hatton, 2007). A precarious economic situation and lower levels of parenting education (Zheng et al., 2012) may affect the development of children with IDs by limiting access to the resources they need (Saunders et al., 2015) and restricting their use of care services (Wu et al., 2003).

### Parental Factors: Parents and Family Well-Being

Research has suggested that having a child with ID may produce negative reactions in the family, and may make family members reluctant to foster the child’s development (Byrne and Cunningham, 1985; Hauser-Cram et al., 2001; Blacher and Baker, 2002; Hastings, 2003). Many parents caring for children and toddlers with a disability report high levels of anxiety, depression, and stress (Dyson, 1997; Keller and Honig, 2004; Al-Qaisy, 2012). It has been found that parents of children with ID generally have higher levels of anxiety, depression, and stress than parents of typically developing children (Olsson and Hwang, 2002; Eisenhower et al., 2005; Oelofsen and Richardson, 2006; Singer, 2006; Baker et al., 2010; Hayes and Watson, 2013). However, not all family members respond in the same way to being a relative of a child with ID. Most research in this area has focused on the well-being of mothers, but very little has been published about the well-being of fathers (Glidden and Natcher, 2009; MacDonald et al., 2010).

Many families with children with disabilities also report lower levels of marital satisfaction (Brobst et al., 2009) and Family Quality of Life (FQoL) (Hu et al., 2012). The literature has shown that social support, especially spousal support, is a protective factor against stress and depression among mothers caring for typically developing children (Boyd, 2002; Holloway et al., 2005; Suzuki et al., 2009; Manuel et al., 2012; Skipstein et al., 2012). Spousal emotional support is also very important for mothers who care for children with ID (Dunst et al., 1986; Glidden and Schoolcraft, 2007). Cohen et al. (2013, 2016) showed the benefits of spousal support for mothers with children with disabilities in relation to child behavior problems. Despite the move toward “family-centered” service models, in the majority of cases it is mothers who spend the largest amount of time caring for the needs of a child with a disability. Mothers of children with disabilities typically tend to be more involved in caregiving and housekeeping than fathers (Pozo, 2010). This caregiving role was found to generate both positive and negative perceptions among mothers of children with ID (Vilaseca et al., 2014). These latter authors established that mothers presented higher levels of anxiety and depression than did fathers from the same family, similar to other studies on families with children with disabilities (Hastings, 2003; Saloviita et al., 2003; Hastings et al., 2005a,b), and they found a significant relationship between the degrees of maternal anxiety and depression and those of fathers (Vilaseca et al., 2014).

Few studies have directly examined the relationship between familial emotional well-being and children’s developmental outcomes. Most have studied the relation between parents’ well-being and children’s behavioral problems (Hauser-Cram et al., 2001; Hastings, 2003; Eisenhower et al., 2005; Cohen et al., 2013, 2016). The increasing understanding of environmental influences on child developmental outcomes stresses the importance of a good environment (Kingston et al., 2012). Some factors of this environment may be less beneficial for infant development, for example, maternal distress or anxiety or depression levels in parents. The detrimental effect of maternal depression after childbirth on emotional, cognitive, and language development in normal developing children is well documented (Cornish et al., 2005; Sohr-Preston and Scaramella, 2006). Some studies have also suggested that maternal anxiety in the postpartum period is associated with lower cognitive and language development scores in typically developing children (Reilly et al., 2006; Glasheen et al., 2010). Another aspect to consider is that maternal distress and parents’ psychological disorders could negatively affect the mother–child interaction, which, in turn, might have an influence on child cognitive and linguistic development (Dilworth-Bart et al., 2007; Feldman and Eidelman, 2009; Edwards and Hans, 2015). However, not all parents of children with disabilities will engage in non-optimal parenting. Several studies have analyzed mothers and fathers of children with disability in different contexts, and have found that parents with positive perceptions of their child with disability expressed more feelings of happiness, family togetherness and personal growth than those with negative perceptions. Increasing parents’ knowledge about disability and developing more coping strategies and positive perceptions led to an increase in family well-being, which helped foster their children’s development. Families who view their relationship with their infant with a disability as something positive experience less parental stress and perceive themselves as more competent parents (Glenn et al., 2009; Ferrer et al., 2016).

### Parental Factors: Parenting

A responsive environment that includes a positive interaction between parents and children, is predictive of better children’s developmental outcomes for infants with ID (Spiker et al., 2002; Innocenti et al., 2013). A wide range of different outcomes may be affected, including neurological development, linguistic, socioemotional, motor, cognitive, and behavioral development, psychopathology and school adjustment (Totsika et al., 2014). Although a large number of studies support the relationship between positive parental practices and child development for typically developing children (Love et al., 2005), fewer studies have examined this association for families with a child with developmental disabilities (for a review, see Dyches et al., 2012). However, this is a very important area of research due to the many stressors these parents experience, and their possible effect on the quality of the parent–child interactions (Gray, 2006; Kersh et al., 2006; Dabrowska and Pisula, 2010).

“Positive parenting” refers to the adult behaviors that promote development in face-to-face interactions in daily routines (Roggman et al., 2013a). Parenting that supports children’s early development can help children with ID to improve their development of language and communication, their cognitive development and their autonomy. Parenting behaviors cover the domains of warmth, responsiveness, encouragement and cognitive stimulation or teaching (Bernier et al., 2010; Roggman et al., 2013a).

Emotional warmth, or affection, refers to the expression of positive emotions, positive evaluation of the child, and positive regard (Roggman et al., 2013a). Emotional warmth and affection are relevant for the construction of secure attachment, which is related in turn to the child’s general development, both for typically developing children and for children with disabilities (Pipp et al., 1992; Kochanska, 2001; Sanders et al., 2004). Several studies have shown that parental affection is associated with less antisocial behavior, and better cognitive abilities and readiness for school (Laible et al., 2000; Zhou et al., 2002; Caspi et al., 2004). Maselko et al. (2011) found that parental affection in the early stages of life also had a positive impact during adulthood.

Responsiveness is another key dimension in the definition of positive parenting. In the context of child–adult interactions, parental behaviors are considered responsive when the adult responds to the signals of the child quickly (immediately) and contingently (in a way linked to the child’s action), adjusting to the child’s initiative and interests, as well to his/her focus of attention and/or action (Spiker et al., 2002; Roggman et al., 2013a; Tamis-LeMonda et al., 2014). Responsiveness has been linked to secure attachment, linguistic development, executive function and general cognition, self-regulation, empathy, and socially appropriate behavior (Osofsky and Thompson, 2000; Landry et al., 2001, 2006; Tamis-LeMonda et al., 2001, 2014; Crouter and Head, 2002; Davidov and Grusec, 2006; Hirsh-Pasek and Burchinal, 2006; Bernier et al., 2010). Both mothers and fathers can be sensitive and supportive to their children (Cabrera et al., 2007). Many studies have demonstrated that fathers who interact with their children in a positive manner and are attentive and responsive promote cognitive, linguistic, social and emotional development in their children, complementing the effects of mothers’ parenting (Shannon et al., 2002; Tamis-LeMonda et al., 2004; Shimpi and Huttenlocher, 2007). Landry et al. (2001) pointed out that parental responsiveness is particularly relevant during early developmental stages.

Parental responsiveness has also been shown to be predictive of developmental outcomes in children with disabilities (Warren and Brady, 2007). In the case of children with intellectual disabilities (ID), maternal responsiveness has been positively related to the child’s linguistic development (Yoder and Warren, 1998, 2000, 2001; Hauser-Cram et al., 2001), cognitive development (Waserman et al., 1985), and social development (Girolametto et al., 1994; Mahoney and Perales, 2003).

Encouragement is another relevant factor defining parental interactions that promote the child’s development. Encouragement is identified in parental behaviors that promote a degree of autonomy, are adjusted to the child’s competencies, set limits and demand maturity according to age, and it predicts adaptive child development and low levels of externalizing problem behavior (Barber, 1996; Hart et al., 2003). Encouraging and supporting children’s efforts, initiative and exploration, while offering appropriate guidance without being intrusive, enhances children’s willingness to take on challenging tasks and develops their executive function, sustained attention and emotion regulation, social adjustment, and adjustment to school (Landry et al., 1997; Hubbs-Tait et al., 2002; Joussemet et al., 2005; Bernier et al., 2010, 2012; Graziano et al., 2011; Roggman et al., 2013a; Fay-Stammbach et al., 2014). In contrast, intrusive parenting has been related to poor social abilities (Rubin et al., 2002; Degnan et al., 2008). Some studies have pointed out the relationships between parental behavior regulation and the developmental outcomes of children with disabilities. Hughes and Kasari (2000) found that children with Down syndrome expressed less pride when they completed a task when their mothers were directive. Maternal directiveness is negatively associated with developmental delays in communicative behaviors (Girolametto and Tannock, 1994). On the other hand, encouraging and supporting the child’s efforts increases their play with objects, the quality and complexity of play, intentional communication, and vocalizations (Cielinski et al., 1995; Roach et al., 1998). Even though these studies have been conducted with mothers, other researchers focusing on fatherhood have shown a relation between the father’s support of the child’s autonomy and the child’s level of executive functioning during the preschool years (Meuwissen and Carlson, 2015). These results are similar to those of previous research with mothers (Sethi et al., 2000; Bernier et al., 2010, 2012).

Finally, cognitive and linguistic stimulation (e.g., explanations, asking the child questions, using a rich vocabulary, joint attention, promoting the child’s participation in adult–child conversation) has been related to cognitive, linguistic, and socioemotional development as well as to emergent literacy skills in typically developing children (Tamis-LeMonda et al., 2001; Hubbs-Tait et al., 2002; Kim-Cohen et al., 2004; Bingham, 2007; Farah et al., 2008). Cognitive and linguistic stimulation focuses on children’s early learning of vocabulary and is predictive of long-term academic success (Cook et al., 2011). In this respect, the father’s role is also relevant for a child’s development (Tamis-LeMonda et al., 2004; Cabrera et al., 2011). In children with disabilities or in children at risk, a developing parenting approach through home visits is useful to engage parents by using specific strategies to promote the child’s language and cognitive development (Peterson et al., 2013; Roggman et al., 2016a,b). Home visiting practices have shown experimental evidence of increased language development and other enhanced developmental outcomes in children with disabilities or with developmental delay using different intervention programs such as the bookmaking intervention, which includes observations of shared conversation and play and cognitive and linguistic stimulation (Boyce et al., 2010a,b, 2017). For families with a child with a disability, achieving good parenting and positive parent–child interactions, such as those described above, can be a real challenge. Children with ID may provide less salient cues, be less responsive or have behavioral problems compared to typically developing children (Innocenti et al., 2013); they may show less emotional expressiveness, and have difficulties in joint attention, language and communication, and behavioral problems, all of which may contribute to difficulties in establishing good interaction patterns (Biringen et al., 2005; Spiker et al., 2005; Salisbury and Copeland, 2013).

Few studies have addressed the relation between diverse variables related to parents and family (demographic variables, quality of parental interactions or parenting, parents’ emotional well-being, family functioning and conjugality) and infant cognitive, linguistic, and motor development, particularly in children with ID. We agree that the transactional model offers a theoretical framework for understanding the impact of parenting on cognitive and language development (Sameroff and Chandler, 1975; Sameroff and Fiese, 2000), considering mothers, fathers and children as units of a family system, with interconnected patterns of actions and relationships. As Cabrera et al. (2011) suggested maternal and paternal parental behaviors are linked to a child’s developmental outcomes, implying a complementary system of parenting, with both commonalities and differences between mothers and fathers within a systemic framework. This is why we consider it is necessary to look at both mothers and fathers when analyzing parenting and other variables in the familial context with respect to child development. Both the child and the parents affect each other in reciprocal ways and the results of this interaction pave the way for subsequent development (Warren and Walker, 2005).

More research in this area is clearly needed, especially because the results could be used to design strategies for families with children with IDs. Our study is clearly exploratory in nature, aiming to examine the relation between family-related demographic variables (e.g., parents’ educational level, family income, and so on), parental factors, and cognitive, linguistic and motor development in young children with IDs. Under “parental factors” we include both the parents’ and family’s well-being (anxiety, depression, parental stress, conjugality, family functioning) and parenting, defined in terms of affection, responsiveness, encouragement, and teaching.

## Materials and Methods

### Participants

Participants were recruited from several Early Intervention Centers (EIC) in Spain. The following criteria were used for inclusion of children in the study: (a) children aged between 20 and 47 months; (b) with an ID (associated or not with another type of disability) diagnosed at least 6 months before carrying out the study.

The study sample comprised 89 children, 61 males (68%) and 28 females (32%), aged from 20 to 47 months (*M* = 33.4, *SD* = 6.8). Fifty-six percent of children were younger than 3 years old (20–35 months), and 44% were 3 years old or over (36–47 months). The degree of ID was mild (from 33 to 64%) in 44%, moderate (from 65 to 74%) in 48% and severe (>75%) in 8%. In Spain, the assessment of the percentage of disability is a standardized process carried out by a governmental agency, the Valuation and Guidance Services for People with Disabilities (CAD). In the case of ID, it is graded as mild, moderate and severe. The centers carry out the assessment and establish the degree of disability. Table 1 contains additional demographic information on the participants.

**Table 1:** Sociodemographic characteristics of the participants.

Characteristic	*N*	%	Characteristic	*N*	%
Child age (20–47 months): *M* (*SD*)	33.4	6.8	Child gender (male)	61	68.0
Mother’s age (27–45 years): *M* (*SD*)	37.0	4.1	Father’s age (26–60 years): *M* (*SD*)	38.9	4.9
Mother’s civil status			Father’s civil status		
Married or cohabiting	79	90.8	Married or cohabiting	78	96.3
Single/divorced/separated/widowed	8	9.2	Single/divorced/separated/widowed	3	3.7
Mother’s educational level			Father’s educational level		
Elementary schooling	16	18.4	Elementary schooling	21	26.6
High school	35	40.2	High school	29	36.7
University degree	36	41.4	University degree	29	36.7
Mother’s employment			Father’s employment		
Full-time job	46	52.9	Full-time job	72	88.9
Partial-time job	24	27.6	Partial-time job	2	2.5
Unemployed or housework	17	19.5	Unemployed or housework	7	8.6
Monthly family income			Received help at home in the care of the children (yes)	47	52.8
Less than €1.314	24	27.9	Satisfaction (0–10) with the services received by their child: *M* (*SD*)	8.7	1.5
€1.314 – €2.450^∗^	25	29.1			
More than €2.450	37	43.0			


*^∗^Considered an average income in Spain (INE-Instituto Nacional de Estadística, 2017).*

### Instruments

A brief *sociodemographic questionnaire* (see Appendix in Supplementary Data Sheet) was produced to collect data from participants (mother, father, and children).

The Spanish version (Caro and Ibáñez, 1992) of the *Hospital Anxiety and Depression Scale* (HADS; Zigmond and Snaith, 1983) was used to assess anxiety and depression symptoms in mothers and fathers. The HADS is a self-reporting screening questionnaire composed of 14 items scored on a four-point Likert-type scale (0–3). Seven items assess depression and seven assess anxiety. Previous research on members of families with children with ID has shown that the HADS is a reliable instrument, with Cronbach’s alphas of at least 0.80 for both anxiety and depression for mothers and fathers (Hastings, 2003). In the Spanish version of the HADS, a factor analysis showed a clear two-factor structure for all groups, and the results demonstrated the internal consistency and reliability of the questionnaire (Quintana et al., 2003). With regard to validity, the correlations with related constructs are acceptable or highly acceptable (Terol-Cantero et al., 2015). In our sample, the HADS Cronbach’s α value for mother’s and father’s anxiety were 0.88 and 0.77, respectively, and 0.80 and 0.78 for mother’s and father’s depression.

The Spanish version (Oronoz et al., 2007) of the *Parental Stress Scale* (PSS; Berry and Jones, 1995) was used to evaluate the degree of stress of the mothers and fathers. The PSS was designed to measure the degree to which situations in one’s life are appraised as stressful. It is a self-report scale, composed of 12 items scored on a five-point Likert scale, from 1 (total disagreement) to 5 (full agreement). The items describe feelings and perceptions about the experience of being a parent. The Spanish version of the PSS demonstrated adequate psychometric properties with high reliability coefficients (internal consistency, α = 0.81, and test–retest, *r* = 0.73), and good validity. In our sample, Cronbach’s α values for both mothers and fathers were 0.82.

The Conjugality subscale on the *Basic Family Relations Inventory* (BFRI; Ibáñez et al., 2012) was used to score the quality of the couple’s relationships. This subscale contains 14 items, which are answered separately by the mother and the father, and refer to how each person perceives the quality of the interaction with his/her partner and the support received from him/her. Items are scored on a five-point Likert scale, from 1 (never) to 5 (always). Conjugality is considered to consist of two poles: harmonious (seven items) and non-harmonious (seven items). As for the subscale’s psychometric properties, the 14-item scores have shown high internal consistency (α = 0.95), reflecting good reliability (Ibáñez et al., 2012). In the present study, the α coefficients were 0.91 for mothers and 0.90 for fathers.

The Spanish version (Fernández-Ballesteros and Sierra, 1989) of the *Family Environment Scale* (Moos and Moos, 1981) was used to measure perceived family interactions. For this study, we used only the Relationships dimension of the FES which consists of 27 true/false items. This dimension is assessed through three subscales: Cohesion (help and support between family members), Expressivity (expression of feelings), and Conflict (openly expressed in the family). The Spanish adaptation of the scale has revealed sufficient rates of reliability and validity (Fernández-Ballesteros and Sierra, 1989). In this study, alpha reliability coefficients were 0.78 for Cohesion, 0.44 for Expressivity, and 0.55 for Conflict. As in previous studies (Adam et al., 2010) the two last subscales yielded values below the desirable levels.

The *Parenting Interactions with Children: Checklist of Observations Linked to Outcomes* (PICCOLO; Roggman et al., 2013b) was used to assess parent–children interactions. PICCOLO is an observational measure of parenting interactions composed by 29 items, scored according to their frequency as 0 (absent), 1 (barely: brief, minor or emerging behavior) and 2 (clearly: definite, strong or frequent behavior). These items measure four interaction dimensions: (a) Affection (expression of affection, positive emotions, positive evaluation of the child and positive regard); (b) Responsiveness (reacting in a sensitive manner to a child’s cues, expressions of needs or interests and behaviors); (c) Encouragement (parents’ support of children’s efforts, exploration, autonomy, choices, creativity, and initiative); and (d) Teaching (includes cognitive and linguistic stimulation, i.e., explanations about causal relations, talk about objects characteristics and questions). The instrument’s reliability is good (see Roggman et al., 2013a) and it shows construct and predictive validity for parents who have a child with a disability (Innocenti et al., 2013). The Spanish validation of the PICCOLO (Vilaseca et al., 2019) found a high interrater reliability; the intraclass correlation coefficients (ICC) ranged from 0.69 for the responsiveness domain to 0.85 for the total score. With respect to internal consistency reliability, all domain and total scores showed satisfactory Cronbach’s alpha coefficients (0.65 for Affection, 0.75 for Responsiveness, 0.76 for Encouragement, 0.72 for Teaching, and 0.88 for the total score). In this study, 25% of mother–child interactions and 21% of father–child interactions were coded by two trained observers; interrater reliability scores were adequate and the ICC ranged from 0.62 to 0.86. Regarding the internal consistency reliability of the total scale, Cronbach’s α values were 0.89 for mothers and 0.90 for fathers. With respect to PICCOLO subscales, Cronbach’s α values for mothers and for fathers were (respectively) 0.56 and 0.59 for Affection; 0.81 and 0.84 for Responsiveness; 0.80 and 0.83 for Encouragement; and 0.68 and 0.63 for Teaching.

Child development was assessed using the Spanish version of the *Bayley Scales of Infant Development-III* (BSID-III; Bayley, 2015). BSID-III scales (Bayley, 2006) are widely used to assess infant and child development between 1 and 42 months of age. Cognitive, Language (Receptive and Expressive communication subtests), and Motor scales (the Fine and Gross Motor subtests) were applied. In the English language version (Bayley, 2006), the mean reliability coefficients were calculated using Fisher’s z transformation. Across all ages, mean stability coefficients were 0.80 or higher. The mean reliability coefficients for the special groups included in the sample were all greater than 0.94. In this study, the Spanish adaptation of the Bayley-III Scales was used and the direct scores were transformed to Spanish percentile scores (Bayley, 2015). BSID-III scales include the possibility of using the instrument to assess children at ages outside the age range of the scale, for example, older children with limited abilities and low developmental age (Bayley, 2015).

### Procedure

First, ethical approval was obtained from the University of Barcelona’s Bioethics Commission (CBUB), in accordance with the International Ethical Guidelines for Health-related Research involving Humans prepared by the Council for International Organizations of Medical Sciences (CIOMS) in collaboration with the World Health Organization (WHO), and the WMA Declaration of Helsinki – Ethical Principles for Medical Research involving Human Subjects.

Secondly, in order to recruit participants we contacted the Catalan Association of Early Intervention, an association that manages the majority of Early Intervention Centers (EIC) in Catalonia. With their consent, 36 EICs in the province of Barcelona and Tarragona were contacted by letter and telephone, informed of the project and invited to participate. Twenty-three EICs accepted the invitation. These results show an acceptable level of participation, since research participation in Early Intervention in Spain promoting collaboration between families and professionals is qualified as moderate (Dalmau-Montala et al., 2017).

After agreeing to participate, the coordinators of the EICs were contacted to request their collaboration in recruiting families based on the inclusion criteria mentioned above. These coordinators informed us of families that might be suitable candidates for the study. Families were informed that their participation would be entirely voluntary and anonymous, and that they would not receive any incentive to participate in the study. To preserve confidentiality, each EIC was given documentation for the candidate families in a sealed envelope. It contained a newsletter, an informed consent form, a sociodemographic questionnaire, and the rest of the instruments cited below (in duplicate) to be completed separately by mother and father, and the FES to be answered jointly. Finally, a brief guide of how to video record the parent–child interaction was included; mothers and fathers were asked to engage separately in a video-recorded of an approximately 10-min play session with their infants at home, with the following instruction “*Interact and play with your children as you typically do.*” The parents had to play with their children using toys they had at home in a natural way, and recorded the video themselves. Seven video recordings were excluded (either because only the child appeared on the tape, because the audio was not clear enough or because they were recorded at bathtime or mealtimes). The PICCOLO (Roggman et al., 2013b) was then used to score parent–child interactions in these video recordings.

The *Bayley Scales of Infant Development-III* (Bayley, 2015) were administered at the EIC by researcher. The parents were present throughout the assessment.

### Data Analysis

Data analyses were conducted in several steps. Firstly, a bivariate analysis was performed to study the relationship between each of the sociodemographic factors and the children’s Bayley percentile scores. For categorical factors, mean Bayley scores were compared via Student *t*-test (for comparing two independent means) or via robust Brown–Forsythe ANOVA (for more than two independent means), followed by *post hoc* Games-Howell’s test for pairwise comparisons. Relationships between continuous sociodemographic factors and Bayley’s scores were analyzed via Pearson product-moment correlation coefficients.

Secondly, the relationship between parental factors and children’s Bayley scores was examined using Pearson correlation coefficients. Finally, variables whose effect was found to be statistically significant (*p* < 0.05) in the previous bivariate analyses were included in a multiple linear regression model to predict child development scores. For variable selection, a stepwise criterion was applied (probability of *F* ≤ 0.05 to add a predictor, and probability of *F* > 0.10 to remove it from the model).

IBM SPSS (version 24.0 for Windows) was used for all statistical analyses. Missing data were handled by pairwise deletion.

## Results

### Sociodemographic Factors and Children’s Developmental Outcomes

Table 1 shows statistical descriptives of the participants’ sociodemographic characteristics. The relationship between each of the factors included in the *sociodemographic questionnaire* (see Appendix in Supplementary Data Sheet) and children’s developmental outcomes (BSID-III percentile scores) was analyzed. In particular, the following sociodemographic factors were included in the study: child’s age and gender; parents’ age, civil status, educational level, employment situation, monthly family income, and degree of satisfaction with the services received by their child (from 0 to 10 points) at the Early Intervention Centers. Parents were also asked whether they received help at home with their children from other individuals or relatives.

Results showed a statistically significant effect of the mother’s educational level on linguistic development [Brown-Forsythe’s *F*(2,56.3) = 5.46; *p* = 0.007; η^2^ = 0.10]. In this regard, the highest mean score for linguistic development was found in children whose mothers had a university degree (*M* = 15.1, *SD* = 2.5), followed by those whose mothers had only elementary schooling (*M* = 8.21, *SD* = 2.0), or had completed high school (*M* = 4.5, *SD* = 0.8). Pairwise comparisons showed higher linguistic development in children whose mothers had a university degree than in those whose mothers had only completed high school (*p* < 0.05); no differences were found between the other categories of the variable.

Using Cohen (1988) benchmarks for interpreting effect sizes, the effect of the “mother’s educational level” on linguistic development can be considered as medium (0.06 ≤ η^2^ < 0.25). None of the sociodemographic factors studied had a significant effect on the children’s cognitive and motor skills.

### Parental Factors

Different parental factors (mother’s/father’s anxiety, depression, stress and conjugality; and familial functioning) were assessed using self-administered questionnaires. Pearson’s correlation coefficients between these parental factors and the children’s scores on the Bayley scales of infant development were computed (Table 2). None of these parental factors was significantly related to cognitive or motor development (*p* > 0.05). However, linguistic development was negatively correlated with mothers’ anxiety and depression scores. This result means that linguistic development was higher in those children whose mothers demonstrated lower anxiety and depression levels.

**Table 2:** Pearson’s correlations between parental scores and children’s BSID-III scores.

	BSID-III outcome					
	
	Cognitive		Language		Motor skill	
			
Parental score	*r*	(*p*)	*r*	(*p*)	*r*	(*p*)
HADS					
Mother anxiety	-0.193	(0.070)	-0.263	(0.013)*	-0.129	(0.284)
Father anxiety	-0.188	(0.096)	-0.210	(0.061)	-0.069	(0.588)
Mother depression	-0.090	(0.404)	-0.234	(0.028)*	-0.068	(0.577)
Father depression	-0.167	(0.140)	-0.197	(0.081)	0.040	(0.756)
PSS				
Mother stress	0.061	(0.574)	-0.088	(0.419)	-0.097	(0.420)
Father stress	0.082	(0.481)	0.032	(0.784)	-0.026	(0.842)
BFRI				
Mother conjugality	0.095	(0.389)	0.120	(0.275)	-0.006	(0.964)
Father conjugality	-0.028	(0.807)	0.049	(0.672)	0.030	(0.817)
FES				
Cohesion	0.051	(0.649)	0.059	(0.594)	0.063	(0.617)
Expressivity	0.021	(0.851)	0.115	(0.300)	0.033	(0.796)
Conflict	-0.112	(0.314)	-0.119	(0.284)	-0.167	(0.185)


*^∗^*p* < 0.05. BSID-III, Bayley Scales of Infant Development-III; HADS, Hospital Anxiety and Depression Scale; PSS, Parental Stress Scale; BFRI, Basic Family Relations Inventory; FES, Family Environment Scale.*

Parenting interactions with children were assessed using an observational measurement instrument (PICCOLO). Statistically significant Pearson’s correlation coefficients were found between cognitive development scores and several PICCOLO domain scores: mother’s responsiveness, father’s responsiveness, and father’s teaching. Likewise, linguistic development scores were positively correlated with mother’s responsiveness and father’s teaching (see Table 3).

**Table 3:** Pearson’s correlations between parenting scores and children’s BSID-III scores.

	BSID-III outcome					
	
	Cognitive		Language		Motor skill	
			
PICCOLO score	*r*	(*p*)	*r*	(*p*)	*r*	(*p*)
Mother				
Affection	-0.054	(0.621)	-0.027	(0.802)	-0.125	(0.310)
Responsiveness	0.312	(0.003)**	0.318	(0.003)**	0.079	(0.522)
Encouragement	0.177	(0.104)	0.174	(0.109)	-0.031	(0.802)
Teaching	0.117	(0.284)	0.176	(0.105)	-0.006	(0.959)
Total	0.193	(0.074)	0.220	(0.042)*	-0.012	(0.921)
Father				
Affection	-0.127	(0.291)	-0.092	(0.444)	-0.071	(0.608)
Responsiveness	0.274	(0.021)*	0.221	(0.064)	0.027	(0.844)
Encouragement	0.216	(0.070)	0.198	(0.098)	0.083	(0.546)
Teaching	0.370	(0.002)**	0.369	(0.002)**	0.081	(0.557)
Total	0.261	(0.028)*	0.244	(0.041)*	0.049	(0.725)


*^∗^*p* < 0.05, ^∗∗^*p* < 0.01. BSID-III, Bayley Scales of Infant Development-III; PICCOLO, Parenting Interactions with Children: Checklist of Observations Linked to Outcomes.*

Therefore, some aspects of parenting interactions seem to promote children’s cognitive and linguistic development, in particular, mother’s responsiveness and father’s responsiveness and teaching. On the other hand, parents’ affection and encouragement were not significantly related to children’s cognitive, linguistic or motor development. At the same time, none of the four domains of parenting interactions was linearly related to the children’s motor development (*p* > 0.05).

### Regression Models on Bayley Scales of Infant Development

Demographic and parental factors whose effect was found to be statistically significant in the previous bivariate analyses (*p* ≤ 0.05) were included in a multiple linear regression model to predict children’s Bayley scores. In order to predict cognitive development, three potential factors were taken into account: (1) mother’s responsiveness, (2) father’s responsiveness, and (3) father’s teaching. Two of the four potential predictors were selected by stepwise criteria for inclusion in the final model. Results (Table 4) indicate that high cognitive development can be predicted by a linear combination of high scores in the mother’s responsiveness and father’s teaching PICCOLO domains. The regression model accounts for 19.6% of the variance of the Bayley cognitive scale scores (adjusted *r*^2^ = 0.196). No more variables or interactions were added to the model because their inclusion did not bring about a significant improvement in the model’s predictive power (*p* > 0.05).

**Table 4:** Linear regression model on Bayley’s cognitive-development score (*n* = 69).

Variable	*B*	*SE*	β	*t*	*p*
Intercept	-10.37	7.12			
Mother’s responsiveness	1.65	0.64	0.296	2.55	0.013
Father’s teaching	1.55	0.65	0.274	2.36	0.021


*B, regression coefficient; SE, standard error; β, standardized regression coefficient.*

The regression model to predict the children’s linguistic development included three of the five potential factors: (1) mother’s educational level, (2) mother’s anxiety score, (3) mother’s depression score, (4) mother’s responsiveness, and (5) father’s teaching. The results (Table 5) indicate that high linguistic development can be predicted by a linear combination of a low score for mother’s anxiety, and high scores for mother’s responsiveness and father’s teaching. The multiple linear regression model accounted for 29.5% of the variance of the Bayley linguistic development scores (adjusted *r*^2^ = 0.295). The remainder of the potential predictors, including two-factor interactions, were excluded because the model’s predictive power did not improve significantly (*p* > 0.05) when any of them was added. It should be noted that two parenting factors (mother’s responsiveness and father’s teaching) were common to both models. PICCOLO scores for mother’s responsiveness and father’s teaching were found to have a significant positive effect on both the cognitive and linguistic development of their children.

**Table 5:** Linear regression model on Bayley’s linguistic-development score (*n* = 69).

Variable	*B*	*SE*	β	*t*	*p*
Intercept	-1.24	5.29			
Mother’s anxiety score	-0.96	0.30	-0.332	-3.21	0.002
Mother’s responsiveness	1.03	0.40	0.281	2.58	0.012
Father’s teaching	0.88	0.41	0.236	2.14	0.036


*B, regression coefficient; SE, standard error; β, standardized regression coefficient.*

## Discussion

### Sociodemographic Factors and Children’s Developmental Outcomes

The overall aim of the study was to examine the relations between some familial variables and a child’s cognitive, linguistic and motor developmental outcomes when the child has intellectual disabilities. The selected familial variables included various sociodemographic characteristics (e.g., parents’ educational level, family income…), parents’ and family’s well-being (anxiety, depression, parental stress, conjugality, family functioning) and parenting, defined as the characteristics of adult–child interactions in terms of affection, responsiveness, encouragement, and teaching (Roggman et al., 2013b). Both mothers’ and fathers’ parenting were considered.

As mentioned in the “Introduction” section, there is a solid tradition of studies relating SES and children’s developmental outcomes, most of them conducted in the general population. In our study, among all the variables included in the sociodemographic questionnaire described in the “Materials and Methods” section, only maternal educational level was related to children’s development, and only for linguistic development. Our results showed higher linguistic development in children whose mothers had a university degree than in those whose mothers only completed high school (*p* < 0.05). No differences were found between mothers with only elementary school and mothers with higher educational levels, though this latter result may be due to the small size of the group of mothers with only elementary school education (18%). Undoubtedly, more significant relationships could be expected based on previous studies in the general population (Sohr-Preston et al., 2013; Roubinov and Boyce, 2017) but the characteristics of the sample, i.e., caregivers with children with ID, need to be considered. In this case, the observed relationship between maternal educational level and a child’s linguistic development is interesting. When comparing mothers and fathers in our sample, we found a significant difference between mothers’ and fathers’ level of employment: only 53% of mothers were employed full-time, compared with 89% of fathers. Another 28% of mothers were partially employed and 19% of them cared full-time for their children and were fully responsible for housework. So, it is reasonable to assume that, in our sample, mothers were interacting daily with their children with ID for more hours than fathers. The effect of the duration of mother–child interactions on linguistic development would be mediated by the quality of these interactions, in terms of affection, responsiveness, non-intrusive behavior and linguistic stimulation, all of them aspects clearly related to maternal educational level (Davis-Kean, 2005; Bornstein and Manian, 2013) and to the children’s linguistic developmental outcomes, both in typically developing children (Shimpi and Huttenlocher, 2007; Roseberry et al., 2014), slow-to-talk children (Levickis et al., 2018) and children with disabilities (Warren et al., 2010; Sterling et al., 2013; DeVeney et al., 2016). From a practical point of view, as pointed out by other authors (Sohr-Preston et al., 2013), parents with lower educational levels would require special attention in programs addressed at promoting a child’s development – both those in the general population and in families with children with disabilities.

### Parental Factors: Parent and Family Well-Being

Few studies have analyzed the relations between the parents’ and family’s well-being (anxiety, depression, parental stress, conjugality, family functioning) on the one hand, and infant cognitive, linguistic and motor development on the other hand, particularly in children with ID. Interestingly, our results showed that children’s linguistic development was negatively correlated with mother’s anxiety and depression scores, but not with father’s anxiety. As mentioned in the introduction, mothers of children with ID report higher levels of anxiety and depression than fathers in the same family (Hastings, 2003; Hastings et al., 2005a,b; Vilaseca et al., 2014). This is a very interesting result because maternal anxiety, cognition and joint activity in the mother–child interaction emerged as the strongest predictors of infant language performance in a sample of young normal developing children and their mothers with anxiety disorders in the postpartum period (Reck et al., 2018). Those results suggested that maternal anxiety and depression may potentially affect infant language development. A hypothesis to consider in future studies is that the effect of a mother’s anxiety on a child’s linguistic development could be mediated by the influence of anxiety on the quality of the mother–child interaction, and particularly on responsiveness, non-intrusive behavior and linguistic stimulation.

Other studies have analyzed the relationship between mothers’ and fathers’ emotional well-being and socioemotional development in typically developing children, and found that anxiety and depression in mothers but not in fathers were significantly associated with social development in children (Huhtala et al., 2014). As Dilworth-Bart et al. (2007) noted, emotional distress in parents, especially mothers, may interfere with their ability to provide the optimal parenting required to promote their children’s development. Having a child with a disability, such as an ID, may cause higher distress in parents, especially in mothers in the early years, because of their prolonged concerns about the development of their child and because it is usually the mother who cares for the child for most of the time in this crucial period. So, it is evident that factors of the immediate environment such as maternal anxiety and depression may be less beneficial for infant development. Our findings illustrate that the emotional well-being of parents, especially mothers, plays a crucial role in infant development, but this is an aspect that has not been researched in depth in families of children with disabilities. In Spain, a few early intervention practitioners have become aware of the importance of responding to families’ needs and of promoting children’s participation in their natural environments, with a slow movement toward a Family-Centered Approach (FCA) (Mas et al., 2018; Vilaseca et al., 2018). In this regard, our results justify the use of family-centered practices in which early intervention practices should focus on the family unit (rather than solely on the child); they also underline the importance of the strengths and competences of the family, and demonstrate that it is the family that can most effectively foster development and learning through the routines and activities that are pursued in the child’s natural and inclusive environments (McWilliam, 2000, 2010; Dunst et al., 2010). Moreover, our results add important information on the impact of the psychological well-being of mothers and fathers in the same family on the development of children with an established ID.

It should be noted that among all risk factors, maternal anxiety symptoms require particular attention in early intervention practices. Children with ID whose mothers experience recurrent anxiety symptomatology could face more difficulties regarding developmental outcomes. This aspect and the parenting characteristics of both mothers and fathers should be taken into account for early intervention practices. A FCA to intervene in the emotional factors, in mothers, may be a protective factor for child development and for parents’ and family well-being. Early intervention approaches focusing on parenting strengths may be able to build upon and promote the positive coping strategies that parents use and reduce the anxiety generated by caring for a child with special needs, especially in mothers. Empirical evidence shows that the FCA has a positive impact on the child and the family unit; it improves parents’ perceptions of their parenting skills, helps to lower the anxiety and stress that sometimes beset families, and helps parents to establish a better relationship with their children and thus promote their development (see the review by Dunst and Espe-Sherwindt, 2016).

Our findings suggest that maternal anxiety and depression symptoms may be negative factors for language development in children with ID. A family-centered model to intervene in the emotional factors, in mothers, may be a protective factor. Our results imply the need to address parental psychological well-being in families with children with ID so as to improve developmental outcomes especially in language.

### Parental Factors: Parenting

As mentioned in the “Introduction” section, a considerable amount of research has documented the relations between parenting and child development, both in families with typically developing children (Love et al., 2005; Mahoney, 2009; Warren et al., 2010; Blair et al., 2014; Vargas-Rubilar and Arán-Filippetti, 2014) and those with developmental delay and disabilities (Spiker et al., 2002; Innocenti et al., 2013). Our results confirm that early parenting behaviors, such as those measured by the PICCOLO, are associated with cognitive and linguistic outcomes in children with ID, with different patterns of effect for mothers and fathers, as also found in typically developing children (Rivero et al., 2018). Our results confirm that both mothers and fathers contribute to their children’s development in different ways (Cabrera et al., 2008, 2018). For mothers, our results showed that responsiveness is associated with cognitive development and linguistic development in children with ID. This is consistent with the findings of several previous studies (Warren et al., 2010; Innocenti et al., 2013; Roseberry et al., 2014; Tamis-LeMonda et al., 2014). Children whose mothers display more responsive behaviors during the early years achieve better language and cognitive development. Our findings also establish that the father’s responsivity and father’s teaching are related to cognitive development in children with ID. Few studies have analyzed fathers’ contributions to their children’s development, especially in families with children with ID. Our findings are consistent with previous studies relating fathers’ teaching to cognitive and linguistic outcomes in typically developing children (Summers et al., 2006; Duursma et al., 2011; Anderson et al., 2013; Leech et al., 2013). Early positive father–child interaction is important for child development, as mentioned previously (Ramchandani et al., 2012). Programs in early intervention are increasingly recognizing the value of engaging fathers in home visits (Roggman et al., 2002; Lawrence et al., 2013), but few studies have explored the relation between positive parenting in mothers and fathers of the same family unit and developmental outcomes in children with a disability in natural settings. In this study, it was the parents themselves who auto-recorded their interactions at home, and so we believe that this is a highly valuable contribution to the existing literature.

We pay particular attention to the role of the father in teaching because our results stress its importance for promoting cognitive and linguistic development in children with ID. Teaching involves activities that include cognitive stimulation, shared conversation and play, explanations and joint attention, which are all known to be essential for promoting child development (Tamis-LeMonda et al., 2001). However, in children with established disabilities these activities may be difficult to carry out, particularly since maintaining a high level of communication or play with a child with ID can be highly challenging. A substantial number of children with ID have unintelligible speech and difficulties with conversational discourse (Abbeduto et al., 2007). Moreover, some of them may present difficulties in social interaction or symptoms of autism (Bailey et al., 2001). In another of our studies (Vilaseca et al., 2019) teaching was the domain with the lowest score on the PICCOLO. Mothers and fathers of children with disabilities engage in more types of affective behavior (warmth, closeness…) and fewer teaching behaviors (conversation, play, cognitive stimulation…) as is the case in younger normally developing children (Roggman et al., 2013a; Peterson et al., 2014). According to our results, children with ID whose fathers display more teaching behaviors during the early years achieve better language and cognitive development. This finding is particularly relevant, especially with regard to intervention. Since fathers’ support of children’s development should be increased in intervention programs, the PICCOLO behaviors could help early intervention practitioners, as this measure focuses on mothers’ and fathers’ behaviors which can be easily recognized and incorporated in intervention plans (Roggman and Cardia, 2016). The Spanish version of the PICCOLO used in this study (Vilaseca et al., 2019) is suitable for research on parents of children with a disability (Innocenti et al., 2013).

On the other hand, parents’ affection and encouragement were not shown to be related to their children’s cognitive, linguistic or motor development. Although we already know that emotional warmth plays a prominent role in children’s development, most previous studies have related parents’ affection to securing attachment and better social behavior in children (Kochanska, 2001), variables that we did not analyze in our study. This aspect should be taken into account in future studies in families of children with disabilities. At the same time, encouraging and supporting the children’s initiative and exploration has been associated with the development of attention, emotion regulation and good social abilities (Rubin et al., 2002; Roggman et al., 2013a) and also with language (Lillard et al., 2013) and cognitive development (Bernier et al., 2010, 2012). The fact that our sample included very young children, with cognitive levels below their chronological age, may explain our results. Perhaps, in older children, encouragement could have greater implications for child development. An interesting result of our study is that none of the four domains of parenting measured using the PICCOLO was related to the children’s motor development. This is one of the least studied aspects in relation to parenting and child development. And although our results suggest that early parenting predicts language and cognitive developmental outcomes in children with a disability, more research is required in order to examine the contributions of different aspects of parenting to children’s motor development.

Finally, the PICCOLO scores for mothers’ responsiveness and fathers’ teaching domain had a significant positive effect on both the cognitive and linguistic development of their children. Our findings show that cognitive and linguistic development can be predicted by the contribution of both parents, each of whom presents different but complementary profiles that promote child development (Cabrera et al., 2014, 2018). This view is consistent with transactional models of human development which have established that there are multidirectional effects on children’s development beyond the additive contributions of the mother and father (Sameroff, 2010; Fitzgerald and Bradley, 2012).

Our results support the call, made previously by other authors (Cabrera et al., 2000; Cox and Paley, 2003; Lamb and Lewis, 2010) for the inclusion of fathers in research. Both mothers and fathers must be considered in this research, to enable us to look more closely at their similarities, differences and complementarities, and to gain a fuller picture of parenting. Our findings corroborate those of other authors (Meuwissen and Carlson, 2015) who have suggested that including fathers in intervention programs may be more beneficial than working with mothers alone.

### Final Remarks

The present study extends the current literature on parenting and child development in families of children with ID. Nevertheless, the study has several limitations that should be considered when interpreting the results. The first is the selection of the sample. Although participants were recruited at Early Intervention Centers, the fact is that the procedure used to select the participants could have been conditioned by the willingness of families to participate (Hoffman et al., 2006); conceivably, the parents who took part were the ones who were the most informed about child development, and most aware of the importance of parental interactions, or even the most confident about their parenting skills. Similarly, it may be that the parents who were most worried about their child’s development were reluctant to participate. Another limitation is the correlational design, which does not permit us to establish a clear causality. Since this was a cross-sectional study, we need to be cautious about the use of the term “predictor” in the regression analysis. We have to take into account that, in this context, “to predict” means just to estimate Bayley’s scores based on the predictor variable scores (demographic and parental scores), and does not necessarily imply direct causality.

Another limitation to consider is the use of self-administered questionnaires. In future research it would be interesting to conduct semi-structured interviews with all family members (including grandmothers, grandfathers or siblings) in order to extract more information and to examine their different perspectives. It would be of interest to compare these new data with those of our current study.

Future research on mothers and fathers with a child with an ID should include recorded observations of their interactions at older ages and the analysis of the possible continuity of patterns of mother– and father–child interactions, and their links to children’s developmental outcomes in the early school years. Information regarding the long-term impact of parental psychological well-being on the development of their children would also be interesting, given that parental distress decreases as the child grows older (Ferrer et al., 2017).

Finally, it would also be of value to measure both the distribution of housework between the father and the mother and the burden assumed by each parent, in order to study whether high levels of responsibility in family tasks could be related to the levels of parental anxiety and depression.

This exploratory study may contribute to the development of theoretical models to explain the mechanism of the effect of specific factors which can later be tested with larger samples.

## Ethics Statement

Ethical approval was obtained from the University of Barcelona’s Bioethics Commission (CBUB), according to the International Ethical Guidelines for Health-related Research Involving Humans prepared by the Council for International Organizations of Medical Sciences (CIOMS) in collaboration with the World Health Organization (WHO), and the WMA Declaration of Helsinki – Ethical Principles for Medical Research Involving Human Subjects.

## Author Contributions

RV, MR, RB, M-JC, EN-P, CV-V, and FF made substantial contributions to conception and design, and/or acquisition of data, and/or analysis and interpretation of data; and participated in drafting the article or revising it critically for important intellectual content; and gave final approval of the version to be submitted.

## Conflict of Interest Statement

The authors declare that the research was conducted in the absence of any commercial or financial relationships that could be construed as a potential conflict of interest.
