# Supplementary material for: Demographic and Parental Factors Associated With Developmental Outcomes in Children With Intellectual Disabilities
Source: Front Psychol. 2019 Apr 24;10:872. doi: 10.3389/fpsyg.2019.00872 (PMC6491580; doi:10.3389/fpsyg.2019.00872)
Supplement: Supplementary file 1 [file Data_Sheet_1.doc]

Appendix

*Sociodemographic Questionnaire*

General information about child and family

Date:

Data of the CHILD PARTICIPATING in the study:

1. Gender:

- Male
- Female

1. Date of birth: _____________/ _____________ /_____________

3. Degree of disability of the child according to the certification received by the CAD

- Mild (33 - 64%)
- Moderate (65 - 74%)
- Severe (≥75 %)

4. Please rate your satisfaction with the Early Intervention Center Service, from 1 (not satisfied) to 10 (very satisfied). Please mark your valuation with a cross on the line below:

1. 2 3 4 5 6 7 8 9 10

Data on the PARENTS:

*Mother*  *Father*

Age: Age:

Marital status:

- Married/Living as a couple
- Divorced/separated
- Widower
- Single

Marital status:

- Married/Living as a couple
- Divorced/separated
- Widower
- Single

Educational attainment:

- Illiterate
- Elementary school
- High school
- University degree
- Other:

Educational attainment:

- Illiterate
- Elementary school
- High school
- University degree
- Other:

Employment status:

- Full-time job
- Part-time job
- Fully responsible for housework
- Unemployed

Employment status:

- Full-time job
- Part-time job
- Fully responsible for housework
- Unemployed

Questions to be answered TOGETHER:

5. Net family income per month, taking into account all sources (for example, family allowance, benefits, unemployment, pensions, etc.).

- <INPUT NAME=\ Less than 1.314 €
- <INPUT NAME=\ <INPUT NAME=\From 1.314 € to 2.450 €
- <INPUT NAME=\ More than 2.450 €

6. Formal or informal support received at home in the care of your child:

- None
- Yes, extended family (e.g., grandparents).
- Yes, baby sitter/domestic help
